# Variation in mitochondrial function in hypoxia-sensitive and hypoxia-tolerant human glioma cells

**DOI:** 10.1038/sj.bjc.6600087

**Published:** 2002-02-12

**Authors:** M L Turcotte, M Parliament, A Franko, J Allalunis-Turner

**Affiliations:** Department of Oncology, University of Alberta, Edmonton, Alberta, Canada; Department of Experimental Oncology, Cross Cancer Institute, 11560 University Avenue, Edmonton, Alberta, Canada T6G 1Z2; Radiation Oncology, Cross Cancer Institute, 11560 University Avenue, Edmonton, Alberta, Canada T6G 1Z2

**Keywords:** glioma, tumour hypoxia, mitochondria, ATP

## Abstract

We have shown previously that human glioblastoma multiforme cells vary in their ability to survive under hypoxic conditions. Under oxygen limiting conditions, hypoxia-tolerant cells decrease their oxygen consumption rate whereas hypoxia-sensitive cells continue to consume oxygen at a relatively steady rate until the oxygen supply becomes exhausted. We now show that hypoxia-tolerant and hypoxia-sensitive cells exhibit distinct patterns of mitochondrial function in response to hypoxic challenge. Hypoxia-tolerant cell lines retain stable mitochondrial membrane potential and ATP concentration when incubated under oxygen limiting conditions. In addition, hypoxia-tolerant cell lines are consistently more sensitive to a wide spectrum of inhibitors of mitochondrial function than are hypoxia-sensitive cells. In contrast, the hypoxia-sensitive cells are unable to maintain stable mitochondrial membrane potential and ATP levels when incubated at reduced oxygen tension. These results demonstrate significant differences in the mitochondrial function between these two phenotypes and reinforce previous data that suggest a regulatory role for mitochondria in the development of hypoxia tolerance.

*British Journal of Cancer* (2002) **86**, 619–624. DOI: 10.1038/sj/bjc/6600087 www.bjcancer.com

© 2002 Cancer Research UK

## 

Glioblastoma multiforme (GBM) is the most common brain tumour in adults. A pathognomonic feature of GBM is the presence of extensive necrosis, and by inference, hypoxia (Nelson *et al*, 1983; Wilden *et al*, 1987). Unlike normal brain tissue, which is exquisitely sensitive to oxygen deprivation, brain tumour cells can survive in relatively hypoxic microenvironments (Kinter *et al*, 1984). Hypoxic cells are relatively resistant to the killing effects of ionizing radiation and some commonly used chemotherapeutic agents, therefore, tumour cells that have acquired the ability to adapt to low oxygen tensions may contribute to local treatment failure.

Our laboratory has developed model systems to investigate the mechanisms by which tumour cells adapt to variations in oxygen supply. *In vivo* measurement of hypoxia in a variety of human tumours by SPECT imaging of iodoazomycin arabinoside showed an unusual pattern of heterogeneity in GBM tumours specifically (Urtasun *et al*, 1996). Using cell lines derived from human GBM biopsy material and human GBM xenografts, we have shown that regional heterogeneity of oxygen consumption exists in GBMs and that modulation of cellular respiration is an important component of the hypoxia defence mechanisms utilized by GBMs (Parliament *et al*, 1997; Franko *et al*, 1998; Allalunis-Turner *et al*, 1999). However, individual tumours and cell lines derived from them vary considerably in their ability to modulate cellular respiration, with some being more or less proficient than others. For example, the hypoxia-tolerant cell lines M006x and M059K significantly reduce their rate of oxygen consumption when incubated under moderately hypoxic conditions (2 or 0.6% oxygen) (Allalunis-Turner *et al*, 1999). M006 cells also preserve their clonogenic potential after exposure to 4 days of moderate hypoxia (0.6% oxygen). In contrast, hypoxia-sensitive M010b cells maintain a steady oxygen consumption rate despite decreased oxygen availability. Further, the ability of M010b cells to form colonies when incubated under hypoxic conditions is significantly reduced despite the maintenance of a steady rate of oxygen consumption.

Cells isolated from certain normal tissue, e.g., hepatocytes and cardiomyocytes, also show the ability to modulate respiration in response to hypoxic stress (Chandel *et al*, 1995, Chandel and Schamacker, 1999, 2000). In the case of heart and liver cells, biochemical evidence indicates the primary involvement of mitochondria in adaptation to hypoxia. It has been suggested that cytochrome *c* oxidase functions as a putative oxygen sensor that mediates changes in respiration in response to changes in oxygen concentration (Chandel *et al*, 1995, Chandel and Schamacker, 1999, 2000). We hypothesize that GBM cells capable of modulating respiration in response to hypoxia utilize oxygen-sensing mechanisms similar to those identified in hepatocytes and cardiomyocytes. Thus, differences in GBM cellular responses to hypoxia may be mediated by differences in mitochondrial function as opposed to cytosolic mechanisms or differences in mitochondrial mass or biogenesis.

In this study, we compared the response of hypoxia-tolerant and -sensitive human GBM cell lines to challenge with hypoxia as well as agents that inhibit aerobic respiration. We also tested whether demand for energy production under oxygen limiting conditions would result in increased mitochondrial biogenesis or would disrupt mitochondrial membrane potential. The results of these studies show that variations in mitochondrial function exist between hypoxia-sensitive and -tolerant GBM cells in response to chemical as well as hypoxic oxidative challenge.

## MATERIALS AND METHODS

### Cell lines and media

The origin and characterization of the GBM cell lines used in this study have been published previously (Allalunis-Turner *et al*, 1992; Parliament *et al*, 1997). The M010b, M006 and M059K cell lines were derived directly from surgical specimens obtained from patients with GBM. M006x is a cell line derived from M006 passage by through a mouse as a tumour xenograft. M006xLo was obtained by incubating M006x cells at 0.6% oxygen for 2 weeks and culturing the surviving cells. All cell lines were cultured in DMEM/F12 media supplemented with 10% foetal calf serum and 1% L-glutamine and used in experiments at passage numbers less than 40.

### Generation of hypoxia

A de-gassing manifold designed by Dr Cameron Koch was used to generate hypoxia (Koch and Painter, 1975). Briefly, cells were seeded onto 60 mm glass plates and allowed to attach for 24 h at 37°C in a standard incubator (5% CO_2_ in air). The plates were then transferred to aluminium chambers from which air was evacuated and then replaced with 5% CO_2_/balance N_2_ until the desired oxygen tension was achieved. The sealed, airtight canisters were then incubated at 37°C for the required amount of time.

### ATP measurement

Intracellular ATP concentrations were measured using a luciferase-based assay (ATP Bioluminescence Assay Kit, Roche) according to manufacturer's instructions. Briefly, cells in exponential growth phase were trypsinized, seeded onto 60 mm glass dishes (10^5^ cells per dish in 5 ml medium), and then incubated at 37°C for ∼20 h in a standard incubator alone or in airtight chambers with the appropriate percentage of oxygen, 5% CO_2_/balance N_2_. The cells were then rinsed with PBS, trypsinized, counted and transferred to a microcentrifuge tube on ice. The cells were washed once in ice cold PBS, re-suspended, and diluted in cold PBS to give 10^3^ cells ml^−1^. Cell aliquots were then added to boiling Tris buffer (100 mM, pH 7.75, 4 mM EDTA), vortexed, boiled for 2 min and then pelleted by centrifugation (10 000 **g** for 3 min at 4°C). Supernatants (100 μl) were transferred to clean microcentrifuge tubes and were kept on ice while covered with aluminium foil. The luciferase reagent was added in the dark to the cellular extracts and the microcentrifuge tube was then wiped with an anti-static agent and placed into a large glass scintillation vial. Photon counts were obtained over 3 min at 20 s intervals using a Beckman scintillation counter (model LS5801) programmed to record single photon events.

### Flow cytometric analysis of mitochondrial mass and function

Cells were seeded in 60 mm glass plates (2×10^5 ^cells/dish in 5 ml medium) and allowed to attach for 6–8 h. The media was then replaced with fresh media containing either JC-1 (100 μM) or Mitofluor Green (75 nM) (Molecular Probes, Eugene, Oregon), agents which permit live-cell analysis of mitochondrial function and mitochondrial mass, respectively (Reers *et al*, 1995; Kunz-Schughart *et al*, 1997). Following loading of the stains, the cells were incubated at various oxygen tensions for 24–96 h after which they were trypsinized and analyzed by flow cytometry within 30 min.

### MTT assay of mitochondrial function and cell viability

Cells were seeded in 96-well plates (2×10^4^ cells/well) and allowed to adhere for 24 h at 37°C with 5% CO_2_ in air. Drugs to be tested were prepared as stock solutions of 10-fold greater than the published lethal concentration from which a series of doubling dilutions was then added to the cells. These agents, together with their sites of action, are documented in Table 1

**Table 1:**
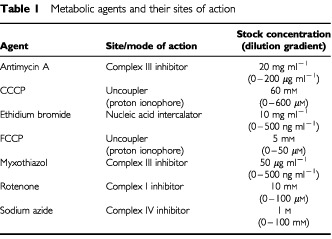
Metabolic agents and their sites of action

. Cells were returned to the incubator for 96 h after which the spent media was replaced with 200 μl of fresh media containing MTT (500 μg ml^−1^) (Cole, 1986; Campling *et al*, 1991). The cells were then incubated for an additional 4 h after which they were lysed with 40% formalin/20% SDS. Cell viability was determined using an ELISA plate reader set to record absorbance at 600–660 nm.

### Data analysis

LC_50_ values were calculated using the GraphPad Prism program. A student *t*-test was used to determine the statistical significance of differences observed.

## RESULTS

### Mitochondrial membrane potential

To study mitochondrial function, live cells grown under various oxygen tensions were labelled with the cell permeant, mitochondria-specific fluorescent probe, JC-1. JC-1 is a lipophilic, cationic, carbocyanine dye which absorbs light at 490 nm and exists as a green fluorescent monomer at cellular concentrations below 0.1 μM. However, an intact mitochondrial membrane potential causes the positively–charged dye to accumulate in the mitochondria where it aggregates to form a red fluorescent dimer. The ratiometric nature of this probe allows detection of changes in mitchondrial membrane potential. Depolarization of the mitochondrial membrane (i.e., a decrease in mitochondrial function) leads to a rapid shift from red (emits at 590 nm) to green fluorescence (emits at 527 nm) as JC-1 ‘spreads out’ from the mitochondria into the cytosol (Reers *et al*, 1995; Kunz-Schughart *et al*, 1997).

This shift from red to green fluorescence is evidenced by the drop in emission in the red range and a corresponding increase in the number of counts per cell registered by the flow cytometer in the green range. The per cent change in membrane potential was thus derived from a comparison of the counts per cell in the red range under normoxic conditions with those of the same cell line under hypoxic conditions ×100.

**Figure 1:**
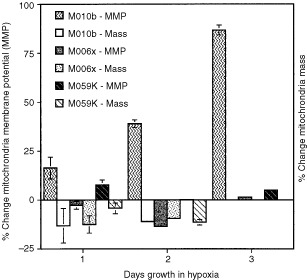
Changes in mitochondrial membrane potential (MMP) (JC-1 fluorescence) and mitochondrial mass (Mitofluor Green fluorescence) in hypoxia-sensitive (M010b) *vs* hypoxia-tolerant (M006x, M059K) cell lines. Cells were grown under normoxic (20% O_2_) or hypoxic conditions (0.6% O_2_) for 1–3 days for MMP determinations, and 1–2 days for mitochondrial mass measurements. The per cent change observed in hypoxic cultures, relative to aerobic controls, is shown. Mean (±s.e.) values calculated for three separate determinations are shown. Where not displayed, error bars were too small to plot.

The hypoxia-sensitive cell line, M010b, showed substantial and consistent increases in mitochondrial membrane potential as determined by JC-1 fluorescence over 3 days growth in an atmosphere of 0.6% oxygen ( Figure 1

). The mean (±s.e.) per cent increases in mitochondrial membrane potential for days 1–3 were 16.4±5.6, 38.9±2 and 86.8±3, respectively. In contrast, hypoxia-tolerant GBM cell lines M006x and M059K showed little change in mitochondrial membrane potential following incubation at reduced oxygen tension. For M006x, the mean (±s.e.) per cent changes in mitochondrial membrane potential for days 1–3 were −2.7±2, −13.5±1 and 2±3, respectively. For M059K, the mean (±s.e.) per cent changes in mitochondrial membrane potential for days 1–3 were 7.7±3, 0.2±1 and 5.1±1, respectively.

### Mitochondrial mass

To test whether hypoxia-tolerance simply reflects the ability to generate more mitochondria, we measured mitochondrial mass in hypoxia-tolerant and -sensitive cells incubated for 1 or 2 days under aerobic or hypoxic (0.6% oxygen) conditions using Mitofluor Green. This cell-permeant, mitochondria selective, fluorescent dye accumulates in mitochondria regardless of mitochondrial membrane potential (Johnson, 1998). No increase in green fluorescence therefore, no evidence of increased mitochondrial biogenesis was observed in any cell line incubated under normoxic *vs* hypoxic conditions. Rather, under hypoxic conditions, mitochondrial mass remained relatively stable, or was slightly decreased (Figure 1). The mean (±s.e.) per cent changes in mitochondrial mass after 1 or 2 days hypoxic incubation were for M010b, −13.2±8.8 and −11.1±0.9; for M006x, −12.5±4.5 and −9.5±.3, and for M059K, −4.3±2.8 and −11.4±1.4.

### ATP measurements

**Figure 2:**
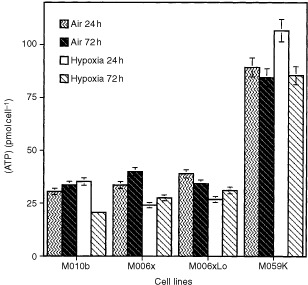
Cellular [ATP] (pmol cell^−1^) in human glioma cell lines maintained under aerobic or hypoxic (0.6% O_2_) conditions for 24 or 72 h. Mean (±s.e.) values calculated for three or more experiments are shown. Where not displayed, error bars were too small to plot.

We tested the ability of human GBM cells to maintain intracellular ATP concentrations when incubated under hypoxic conditions. Figure 2

shows mean values (±s.e.) calculated for three or more replicate experiments. When measured after 24 h incubation in an atmosphere of 0.6% oxygen, the [ATP] of hypoxia–sensitive M010b cells was equivalent to that of aerobic M010b controls. However, M010b cells were unable to maintain a stable [ATP] under prolonged hypoxic conditions, and after 3 days of hypoxia, the [ATP] was reduced to ∼60% of day 3 aerobic control values (33.6±3 *vs* 20.6±2.7; *P*<0.015). Following 24 h of hypoxia, the mean [ATP] of hypoxia-tolerant M006x cells was reduced to 72% of control values (33.5±3.3 *vs* 24.1±4.8; *P*<0.016). However, under continued hypoxic incubation, the mean [ATP] M006x cells increased slightly and after 3 days, was not significantly different from that of M006x aerobic controls. M006xLo cells also showed a decrease in mean [ATP] after 24 h growth in hypoxia, however this difference was not significant. After 3 days of growth in hypoxia, the mean [ATP] values were similar to that of controls. Finally, hypoxia-tolerant M059K were able to maintain stable, or slightly increased [ATP] values throughout the entire hypoxic incubation period. (The large difference in mean [ATP] of M059K cells as compared to the other cell lines is consistent with the fact that M059K cells have an ∼two-fold greater volume than the other cell lines in this study, and would therefore be expected to contain proportionately more [ATP]). Importantly, after 3 days of hypoxia, all of the hypoxia-tolerant cell lines (M006x, M006xLo, M059K) were able to maintain mean [ATP] equivalent to that of the relevant aerobic control. In contrast, hypoxia-sensitive M010b cells were unable to maintain stable [ATP].

### Sensitivity to mitochondrial poisons

To assess the potential involvement of the mitochondrial electron transport chain in hypoxia tolerance, we treated human GBM cells with known chemical inhibitors of mitochondrial function. We utilized specific inhibitors of oxidative phosphorylation as well as general inhibitors of mitochondrial function to distinguish particular pathway disparities (Table 1). The mitochondrial uncouplers, FCCP and CCCP, disrupt mitochondrial membrane integrity but do not affect specific components of the electron transport chain. Ethidium bromide is a nucleic acid intercalating agent. Rotenone is a potent inhibitor of complex I, myxothiazol and antimycin A are complex III inhibitors, and sodium azide specifically inhibits cytochrome *c* oxidase (complex IV). LC_50_ values were calculated for each drug to facilitate comparison of their effects on mitochondrial function. The sensitivities of each cell phenotype (i.e., hypoxia-tolerant *vs* hypoxia-sensitive) was compared by calculating ratios of the M006x LC_50_ values divided by the M010b LC_50_ (standardized as one) for each agent. Ratios of less than one therefore indicate a greater sensitivity of M006x cells to the agent tested as compared to M010b cells. Ratios greater than one indicate an increased resistance to the test agent for M006x cells. As shown in Table 2

**Table 2:**
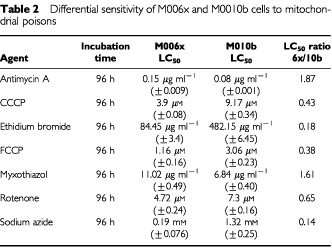
Differential sensitivity of M006x and M0010b cells to mitochondrial poisons

, hypoxia-tolerant and -sensitive GBM cells showed significant differences in their sensitivity to these mitochondrial poisons. In general, hypoxia-tolerant cells were more sensitive to inhibitors of mitochondrial function (CCCP, FCCP, rotenone, sodium azide) or a mitochondrial DNA intercalator, ethidium bromide (which has been used extensively at low concentrations to selectively inhibit mitochondrial vs nuclear DNA) (Chandel *et al* 1999). However, a notable exception to the overall greater sensitivity of hypoxia-tolerant cells was the relatively greater resistance of M006x cells to the complex III inhibitors myxothiazol and antimycin A.

## DISCUSSION

Although all mammals require oxygen for survival, individual organ systems vary in their ability to tolerate hypoxic stress. The normal brain is exquisitely sensitive to oxygen deprivation. In contrast, a large body of evidence indicates that hepatocytes and cardiomyocytes can survive brief periods of hypoxia. For example, hepatocytes exposed to hypoxia (*p*O_2_=20 torr) for 3–5 h show no evidence of cell death (Kinter *et al*, 1984). Similarly, cardiomyocytes have been shown to maintain viability under hypoxic conditions ranging from 2–48 h in duration (Chandel *et al*, 1995; Budinger *et al*, 1996, 1998). Key features of this metabolic adaptation to hypoxia have been identified. They include: (1) the ability to reversibly suppress oxygen consumption under oxygen limiting conditions; (2) the ability to maintain cellular [ATP] throughout the hypoxic period and (3) the ability to maintain a stable mitochondrial membrane potential under hypoxia (Hochachka *et al*, 1996). This suggests that in hypoxia tolerant cells, ATP synthesis and utilization remain closely coupled. In addition, while hypoxia produces no acute change in mitochondrial potential, cytochrome *c* oxidase (complex IV) undergoes a reversible decrease in *V*_max_ (Chandel *et al*, 1995; Budinger *et al*, 1996, 1998). Further, hypoxia does not alter the overall control exhibited by NADH over respiration, suggesting that the mechanism responsible for hypoxia tolerance resides exclusively within the mitochondrial electron transport chain downstream of NADH (TCA cycle) supply.

The results obtained from study of hypoxia-tolerant hepatocytes and cardiomyocytes show interesting parallels to our own work. We have reported previously that cell lines derived from human GBM specimens vary in their ability to modulate oxygen consumption in hypoxic environments. While hypoxia-tolerant cell lines such as M006, M006x and M059K were able to decrease their oxygen consumption as oxygen concentrations declined, oxygen consumption by the hypoxia-sensitive line, M010b, remained constant regardless of oxygen availability (Allalunis-Turner *et al*, 1999). Hypoxia-tolerant hepatocytes and cardiomyocytes exhibit this property also. Here we report that under reduced oxygen tensions similar to those that may be encountered *in vivo*, hypoxia tolerant GBM cells display two additional characteristics of tissues adapted to hypoxia: (1) the stabilization of mitochondria membrane potential and (2) the maintenance of intracellular [ATP]. In addition, we have shown that ability to maintain near aerobic [ATP] was not the consequence of generating more mitochondria *per se*. Thus, key components of the hypoxia defence mechanisms of hypoxia-tolerant normal mammalian tissues are expressed in hypoxia-tolerant GBM cells.

The co-ordinated activity of many different cellular and biochemical mechanisms contribute to the development of a hypoxia-tolerant phenotype. In this study, we examined a putative role for mitochondria in the development of hypoxia tolerance in human GBM cells. Although the molecular basis for a cell's ability to match oxygen utilization to oxygen availability is unknown, it has been established that oxygen sensing in mammals, as well as yeast, likely involves a redox-sensitive haemoprotein. The involvement of the mitochondria in mediating the response to hypoxia has long been postulated. Current evidence points to a component of the oxidative phosphorylation (OXPHOS) complex as an ‘oxygen sensor’. Cytochrome bc1 oxidoreductase (complex III), cytochrome *c* and cytochrome *c* oxidase (COX, complex IV) have all been proposed to play important roles in initial sensing of oxygen levels (Bunn and Poyton, 1996; Semenza, 1999; Wenger, 2000). The hypoxia-induced increase in turnover rate of the COX enzyme in yeast (resulting in an increased efficiency of electron transport), has been attributed to the expression of a hypoxia-specific COX Vb isoform. In mammalian systems, similar tissue-specific variations in specific COX subunits as well as cytochrome *c* have been described and correlated with significant differences in electron transfer kinetics. Furthermore, results of recent studies using transmitochondrial constructs suggest an oxygen sensing mechanism driven by the hypoxia-induced generation of reactive oxygen species (ROS) by complex 3 (Chandel *et al*, 1998, Chandel and Schumacker, 1999, 2000).

The significantly greater stability of the mitochondrial membrane potential in M006x and M059K cells indicates a basic difference in the way these hypoxia-tolerant cell lines react to oxygen deprivation. Whereas the hypoxia-sensitive M010b cells increased mitochondrial proton output over a 3 day exposure to hypoxia, M006x and M059K cells retained a stable membrane potential despite an oxygen deficit. Intracellular [ATP] was also maintained at stable levels in M059K and M006x cells until very low oxygen tensions eventually caused collapse of energy production. In contrast, the response of M010b to hypoxic challenge was to maintain a constant oxygen consumption rate and to increase mitochondrial membrane potential (i.e., mitochondrial proton pumping). However, despite these activities, there was no significant increase in intracellular ATP concentration [ATP]. In fact, after a small initial increase at 24 h, [ATP] dropped significantly. This seeming paradox (i.e., a steady oxygen consumption rate, initially stable [ATP]) leading to an ultimate failure of energy production may reflect large-scale mitochondrial disruption as the inner mitochondrial membrane dismantles causing both the electron transport chain and ATP synthase to become non-functional. In direct contrast, hypoxia-tolerant M006x and M059K cells maintained a stable mitochondrial membrane potential as well as intracellular [ATP] under low oxygen growth conditions, despite a small initial decrease at the beginning of the hypoxic exposure. These results suggest that the rate of ATP utilization is being down-regulated to match decreased production rates as in the Hochachka model (Hochachka *et al*, 1996).

Most ATP is generated through a proton motive force in conjunction with mitochondrial ATP synthase and the proton motive force is itself generated by pumping protons from areas of low to high concentrations. As the energy required to pump these protons is generated through the transfer of electrons in the electron transport chain, our data collectively indicates stabilization of the electron transport chain in hypoxia-tolerant GBM cells. This would entail a constant proton flow in spite of decreased electron transport due to the decreased availability of the final electron acceptor, O_2_. A putative mechanism that may regulate stabilization of the electron transport chain is expression of hypoxia-sensitive isoforms of one or more of the molecules in the electron transport complex as had been described for yeast (Kwast *et al*, 1999). In yeast, it has been shown that COX5b, the hypoxic isoform of COX5, is more efficient at pumping protons across the inner mitochondrial membrane (i.e., requires less energy to pump the same number of protons), thereby producing a stable membrane potential and ATP concentration despite utilizing fewer oxygen molecules. We are currently investigating the possible expression of hypoxic isoforms of OXPHOS proteins in our hypoxia-tolerant GBM cells.

In addition to examining changes in mitochondrial membrane potential, we treated cells with a variety of inhibitors of oxidative phosphorylation and mitochondrial function in an attempt to uncover differences in the sensitivity of the chemiosmotic system of the different cell lines. Our results suggest that fundamental differences exist in the oxidative ‘machinery’ of M006x and M010b. When compared to hypoxia-sensitive M010b cells, hypoxia-tolerant M006x cells were more sensitive to agents that specifically interfere with aerobic respiration. The exception to this was the sensitivity to complex III inhibitors myxothiazol and antimycin A. In repeated trials, M006x cells were consistently less sensitive to these agents than were M010b cells. This difference is evocative of work by Chandel *et al* (2000) that demonstrated a role for complex III generated reactive oxygen species in the stabilization of HIF-1α during induction of hypoxia-responsive genes. It is possible that there is a link between the greater resistance of hypoxia-tolerant cells to complex III inhibitors and the role of complex III in the initiation and stable maintenance of hypoxia-responsive genes which may be responsible for this phenotype. Experiments are presently underway in our laboratory to investigate the nature of these differences as well as to examine ROS generation in these glioma cell lines. Additionally, the possible role of glycolytic and non-mitochondrial determinants of hypoxia tolerance to these phenotypes is also being examined.

Spheroid and xenograft studies suggest that most human and rodent tumour cells are of the hypoxia-sensitive phenotype (Freyer and Sutherland, 1985; Hlatky *et al*, 1989). Radiobiological hypoxia is thought to arise as a consequence of cells continuing to consume oxygen at a steady rate regardless of the existence of a steep oxygen gradient with increasing distance from blood supply (Hystad and Rofstad, 1994; Gulledge and Dewhirst, 1996). If the glucose concentration in regions distal to the blood supply remains adequate, some cells can subsist for a short period by utilizing glycolysis to produce ATP in the absence of oxygen (Freyer and Sutherland, 1985; Dewhirst *et al*, 1994). It is these cells, which exist for a short period of time (usually less than a few hours) in the narrow hypoxic area adjacent to the necrotic centre of a tumour, (where glucose concentration also becomes limiting), that resist the killing effects of ionizing radiation and label with nitroimidazole hypoxia markers.

The effect of hypoxia-tolerant cells on a heterogeneous tumour population may depend on the proportion of such ‘M006x-like’ cells present. If the majority of tumour cells are hypoxia-tolerant, this would result in a shallower oxygen gradient as these cells would reduce oxygen consumption when ‘sensing’ decreased oxygen availability. This, in turn, would permit the diffusion of oxygen further into a tissue and thus decrease or eradicate areas that label as radiobiologically hypoxic. This set of conditions offers a possible explanation for the failure to detect hypoxia in some large breast tumours (Okunieff *et al*, 1993). The existence of hypoxia-tolerant cells in more modest percentages within a larger population of predominantly hypoxia-sensitive cells could also have a clinically relevant impact. Cells with a M006x-like phenotype could exist in an area of the tumour where both oxygen and glucose are limiting, thus extending area of radiobiological hypoxia to the peri-necrotic centre of the tumour. The addition of even a few additional layers of viable cells could have substantial clinical consequences and may, in part, explain the extraordinary radioresistance of glioma tumours in conjunction with their poor hypoxic labelling *in vivo*.

In summary, our results indicate that distinct variations in mitochondrial function exist between hypoxia-sensitive and -tolerant GBM cells in response to chemical as well as hypoxic oxidative challenge. Under normoxic conditions, differences in the OXPHOS systems of hypoxia-tolerant GBM cells distinguish them from M010b cells that express normal brain-like responses to hypoxic challenge. Our models for these studies are human GBM cells that may not completely mirror the response of normal tissues to hypoxic insult. In addition, the hypoxia-tolerant and hypoxia-sensitive phenotypes are broadly defined and their expression may vary from one human tumour to another. However, studies such as these may provide insight into the mechanisms utilized by other types of tumours such as tumours of the cervix, lung, breast and skin that have been shown to contain hypoxic regions.
